# Novel genomic regions on chromosome 5B controlling wheat powdery mildew seedling resistance under Egyptian conditions

**DOI:** 10.3389/fpls.2023.1160657

**Published:** 2023-05-10

**Authors:** Amira M.I. Mourad, Rania M. Hamdy, Samar M. Esmail

**Affiliations:** ^1^ Leibniz Institute of Plant Genetics and Crop Plant Research (IPK), Seeland, OT Gatersleben, Germany; ^2^ Department of Agronomy, Faculty of Agriculture, Assiut University, Assiut, Egypt; ^3^ Food Science and Technology Department, Faculty of Agriculture, Assiut University, Assiut, Egypt; ^4^ Wheat Disease Research Department, Plant Pathology Research Institute, Agricultural Research Center, Giza, Egypt

**Keywords:** genome-wide association study, gene enrichment, biological process pathway, functional annotation pathway, gene network

## Abstract

Wheat powdery mildew (PM) causes significant yield losses worldwide. None of the Egyptian wheat cultivars was detected to be highly resistant to such a severe disease. Therefore, a diverse spring wheat panel was evaluated for PM seedling resistance using different *Bgt* conidiospores collected from Egyptian fields in two growing seasons. The evaluation was done in two separate experiments. Highly significant differences were found between the two experiments suggesting the presence of different isolates populations. Highly significant differences were found among the tested genotypes confirming the ability to improve PM resistance using the recent panel. Genome-wide association study (GWAS) was done for each experiment separately and a total of 71 significant markers located within 36 gene models were identified. The majority of these markers are located on chromosome 5B. Haplotype block analysis identified seven blocks containing the significant markers on chromosome 5B. Five gene models were identified on the short arm of the chromosome. Gene enrichment analysis identified five and seven pathways based on the biological process and molecular functions respectively for the detected gene models. All these pathways are associated with disease resistance in wheat. The genomic regions on 5B seem to be novel regions that are associated with PM resistance under Egyptian conditions. Selection of superior genotypes was done and Grecian genotypes seem to be a good source for improving PM resistance under Egyptian conditions.

## 1 Introduction

Wheat is the most important cereal crop all over the world. It provides food for almost 35% of the world’s population (Paux et al., 2008; Mondal et al., 2021). One of the major devasting foliar diseases that reduce wheat crop and quality is powdery mildew (PM) caused by *Blumeria graminis* f.sp *tritici* (*Bgt*) (Singh et al., 2016; Figureueroa et al., 2018). In favorable conditions, PM infection could cause yield losses of up to 62% in many wheat planting areas (Costamilan, 2005; Maxwell et al., 2009; El-Shamy et al., 2012; Huang et al., 2013). Recently, PM was reported to occur annually in Egyptian fields and is considered more dangerous than rusts that have been effectively controlled (El-Shamy and Mohamed, 2022). Few efforts have been made to control such a serious disease in Egyptian fields. Some studies tested the presence of specific *Pm* genes in the Egyptian wheat germplasm (Emara et al., 2016; Elsayed and Elkot, 2020; Draz et al., 2022). Others figured out the different *Bgt* isolates exist in the Egyptian *Bgt* populations (El-Shamy et al., 2012; El-Shamy and Mohamed, 2022). However, a deep understanding of the genetic control of PM resistance will help wheat breeders to produce highly resistant genotypes.

Like wheat rust diseases, wheat resistance to PM could be classified into two types: seedling resistance (also known as race-specific resistance), and adult plant resistance (also known as race-non-specific resistance). Race-specific resistance is controlled by one gene that has a single effect. Thus resistance could be overcome if a new *Bgt* race appears (Cheng et al., 2022). Therefore, pyramiding many seedling resistance genes in the same genotype could provide a broad-spectrum resistance to such a serious disease. Several *Pm* resistance genes have been identified in wheat. Out of these genes, only 68 genes have been identified and mapped on specific chromosomes of the wheat genome (He et al., 2021). However, some of these genes were found to be associated with deleterious traits (Hurni et al., 2014). Therefore, few of these genes were used widely to improve PM resistance and could be found in modern wheat cultivars. Looking for other resistance genes/genomic regions is required to overcome PM in wheat.

Association mapping (AM) is one of the most effective ways to detect genomic regions controlling specific traits. To perform AM, genome-wide association study (GWAS) could be done using diverse populations. In the recent study, a diverse panel was used to identify other sources of PM resistance. This panel was collected from 22 different countries hence it is appropriate for GWAS. Diverse populations are always helpful in detecting new sources of improving targeted traits (Sallam et al., 2018; Mourad et al., 2021b; Amro et al., 2022). In wheat, GWAS is used widely in detecting marker-trait associations (MTAs) for different biotic and abiotic traits (Noubissié et al., 2017 ; Daba et al., 2018; Liu et al., 2018; Alqudaha et al., 2019; Ward et al., 2019; Kumar et al., 2020; Eltaher et al., 2022; Hasseb et al., 2022; Mourad et al., 2022b). Due to the rapid development of state-of-art DNA sequencing techniques, candidate genes of the wheat genome have been identified. Therefore, identifying gene models harboring significant markers associated with specific traits could be easily detected. Many databases for functional annotation, expression of wheat genes, gene enrichment, and different plant pathways became available online. Some of these databases are *WheatExp* (Pearce et al., 2015), *Knetminer* (Hassani-Pak et al., 2021), *EnsemblePlants* (Kersey et al., 2016), and *ShinyGo* (Ge and Jung, 2018). Combining the information obtained from these databases with the GWAS study will facilitate improving wheat cultivars for different conditions.

The objectives of this study are: (1) understanding the genetic control of PM resistance under Egyptian conditions using a diverse wheat panel, (2) identifying MTAs associated with different *Bgt* race populations collected from the Egyptian fields, and (3) selecting the most promising genotypes that could be used in future breeding programs to improve PM resistance in Egyptian wheat germplasm.

## 2 Materials and methods

### 2.1 Plant materials

In the current study, two sets of plant materials were evaluated for their PM seedling resistance, namely the tested set and differential lines set. The tested set consists of 198 spring wheat genotypes that represent old and new cultivars collected from 22 different countries around the world (**Table S1**). The majority of these tested genotypes are from Egypt. Seeds of the non-Egyptian cultivars were obtained from the USDA-ARS, Aberdeen, ID, United States. However, seeds of the Egyptian cultivars were obtained from the Egyptian governorate. The differential lines set consists of 21 different isolines (IL) that carry 20 different PM resistance genes in addition to one susceptible genotype (**Table S2**). Seeds of the differential lines were obtained from Dr. Abdelrazek Abdelrhim, Minia University, and were used in a previous study (Abdelrhim et al., 2018).

### 2.2 Experimental design, powdery mildew inoculation, and evaluation

All the plant materials were evaluated for their PM seedling resistance in two different experiments, Exp. I and Exp. II. Both experiments were conducted in the greenhouses of the Wheat Disease Research Department, Agriculture Research Station, Egypt. In each experiment, different *Bgt* populations were used as follows: *Bgt1* population was collected from the Egyptian field growing season 2021 and was used in Exp. I., while *Bgt2* population was collected from the Egyptian field growing season 2022 and was used in Exp. II. In each experiment, the experimental design was a randomized complete block design (RCBD) with three replications. In each replication, each tested genotype was sown in plastic pots (10 cm diameter) with 10 kernels/pot. Planting was done in clay soils and the irrigation was applied as recommended.

PM inoculation was artificially done in each experiment by collecting naturally infected plants during the mentioned growing seasons from different commercial wheat fields at different locations in Egypt. The inoculum was multiplied on the susceptible check (Jagalene) by shaking conidia collected from infected plants over the 8-day seedling leaves of healthy plants (Browder, 1971; Leath and Bowen, 1989). The inoculated seedlings were maintained in the greenhouse at 20 ± 2°C, 70–90% relative humidity under a 16 h photoperiod with a light intensity of approximately 14,000 lux meter. To inoculate the tested genotypes, 8 days of seedlings of each genotype were inoculated with 10-days Jagalene infected seedlings using rubbing technique. The inoculated seedlings were maintained in the greenhouse for 12 days until scoring. Infection type (IT) was recorded using MQ et al. (1987)’ scale ranging from 0-4. In this scale, genotypes with ITs ranging from 0–2 were considered as ‘R’ (resistant), while genotypes with ITs ranging from 3–4 were considered as ‘S’ (susceptible).

### 2.3 Statistical analysis of powdery mildew seedling resistance

The recorded ITs were converted from MQ et al. (1987)’ scale to a linear IT (LIT) to convert the phenotypic data from a qualitative to a quantitative scale for analyses. The LIT was the same used by Mourad et al. (2018). In the LIT, genotypes with scores ranging from 0-5 were considered resistant, while genotypes with scores ranging from 6 to 9 were considered susceptible.

Two different ANOVA models were used to analyze PM seedling resistance using PLABSTAT software (Utz, 1997) as follows:


(1) $$Y_{ijk}=\;µ+\;g_{i}+\;r_{j}+\;E_{k}+\;gy_{ik}+\;e_{ijk}$$

where, Y_ijk_ is an observation of genotype *i* in replication *j* which was planted in experiment *k*, µ is the general mean; g_i_, r_i_, and E_k_ are the main effects of genotypes (fixed effects), replications and experiments (random effects), respectively; e_ijk_ is the error. This model was used to identify the differences between the two experiments Exp. I. and Exp. II.


(2) $$Y_{ij}=\;µ+\;r_{j}+\;g_{i}+\;gr_{ij}+\;e_{ij}$$

where Y_ij_ is an observation of genotype i in replication j, μ is the general mean; g_i_, r_j_ are the main effects of genotypes and replications, respectively; e_ij_ is the error. This model was used for each experiment separately to identify the differences between the tested genotypes under each experiment.

Broad-sense heritability was calculated using the following formula:


$$H^{2}=\;\sigma_{G}^{2}/\left(\sigma_{G}^{2}+\frac{\sigma_{GR}^{2}}{r}\right)$$

where $\sigma_{G}^{2}$ and $\sigma_{R}^{2}$ are the variance of the lines and the residuals, respectively. *r* is the number of replicates.

### 2.4 Genotyping of the tested materials

All the tested plant materials (198 genotypes) were genotyped using a 25K Infinium iSelect array (25K-SNPs). This marker set was generated by SGS Institute Fresenius GmbH TraitGenetics Section (Gatersleben, Germany) as described previously in (Aleksandrov et al., 2021; Mourad et al., 2022b). In addition, 103 genotypes from the tested materials were genotyped using genotyping-by-sequencing (GBS) as described in (Mourad et al., 2020; Abou-Zeid and Mourad, 2021; Mourad et al., 2022a). Both marker sets were filtered for minor allele frequency (MAF >0.05), maximum missing sites/SNP<20%, and maximum missing sites/genotypes<20%. A total of 21,093 and 11,362 markers were obtained for 25K-SNPs and GBS, respectively. Both marker sets were reported to cover different parts of the wheat genome (Mourad et al., 2022a; Esmail et al., 2023).

### 2.5 Genome-wide association study (GWAS) for powdery mildew seedling resistance

To identify the genomic regions controlling PM seedling resistance, the best linear unbiased predictors for LIT (BLUPs) were used to conduct GWAS. BLUPs values were calculated for each experiment separately using lme4 R package (Bates et al., 2015). Three different models were used for GWAS analyses; Generalized linear model (GLM), Mixed Linear Model (MLM), and Fixed and random model Circulating Probability Unification (FarmCPU) using rMVP R package (Yin et al., 2021). Furthermore, PCA, kinship (Kin), and PCA+Kin were included and tested in each model. The best model was detected for each evaluation based on the distribution of the expected *p*-values and observed *p*-values in the QQ-plot. The significant markers were identified using *p*-value<0.001 (-log_10_>3.00). The target allele marker was the one that decreased the LIT. The phenotypic variation explained by each marker was calculated using TASSEL 5.0 software (Bradbury et al., 2007).

### 2.6 Haplotype block analysis

For the significant markers located on the same chromosome, haplotype block analysis was performed using Haploview 4.2 software (Barrett et al., 2005). A cutoff value of 1% was used as described by (Mourad et al., 2018). The four-gamete method was used to construct haplotype blocks. In this method, block boundaries are created where evidence of recombination between adjacent SNPs based on the presence of all four gametic types exists (Wang et al., 2002).

### 2.7 Gene models harboring the significant markers, gene enrichment analysis, and network

To provide more understanding of the genetic control of PM seedling resistance, high-confidence gene models that harbor significant markers were identified. To identify these gene models, the position of the significant markers in base pair (bp) was investigated for the gene models that they were located within using *EnsemblePlants* database (https://plants.ensembl.org/Triticum_aestivum/Info/Index .). Furthermore, the functional annotation of the identified gene models was detected using the genome annotation provided by International Wheat Genome Sequence Consortium (IWGSC) v.1.0. The relationship between the functional annotation and disease resistance in wheat was examined using *KnetMiner* database (Hassani-Pak et al., 2021). The gene enrichment of the identified gene models based on biological process, molecular function, and cellular component was investigated using ShinyGo 0.76 database (Ge and Jung, 2018) with a *p-*value<0.10 and visualized using SRPLOT database (http://www.bioinformatics.com.cn/srplot).

## 3 Results

### 3.1 Evaluation of powdery mildew isogenic lines and susceptible checks

To investigate the possible resistant *Pm* genes that are effective against the Egyptian *Bgt* isolates, a set of 20 isogenic lines (IL) carrying 20 different genes were evaluated in both experiments (Exp. I and Exp. II). Out of the 20 IL, four (*NCA6*, *NACG13*, *Pm1b*, and *Pm37*) were very resistant in both evaluations (**Table S2**). Four IL; *MIAG12*, *Pm3b*, *Pm34*, and *Pm36* were found to be resistant (0<IT<2) in both experiments. Only one IL carrying *Pm8* gene was found to be susceptible in both experiments. However, the remaining eleven IL were resistant in Exp. I. and susceptible in Exp. II. To investigate the successes of the artificial infection, Jagalene (susceptible check) was found to be highly susceptible (IT=4) in both experiments (Exp. I. and Exp. II.).

### 3.2 Genetic variation of powdery mildew seedling resistance to the different populations of Bgt isolates in the tested spring wheat panel

Based on the analysis of variance results, highly significant differences were found between the two experiments (Exp. I. and Exp. II.) (**Table 1**). Furthermore, a highly significant genotype x experiment interaction was found. The tested genotypes revealed highly significant differences in their resistance to each population of *Bgt* isolates (**Table S3**). High broad-sense heritability estimates (H^2^) of PM resistance were found in both experiments with a value of 0.99 and 0.93 for Exp. I. and Exp. II., respectively.

**Table 1:** Mean square of powdery mildew seedling resistance in 198-spring wheat genotypes among the two experiments (Exp.I. and Exp.II.).

Source of variance	d.f	MS
Experiments (E)	1	300.03**
Replications (R)	2	0.93
Genotypes (G)	195	23.10**
GE	195	23.02**
GER	782	0.59
Total	1175	–
Heritability	0.97	

^*^P<0.05 ^**^P<0.01.

**Figure 1:**
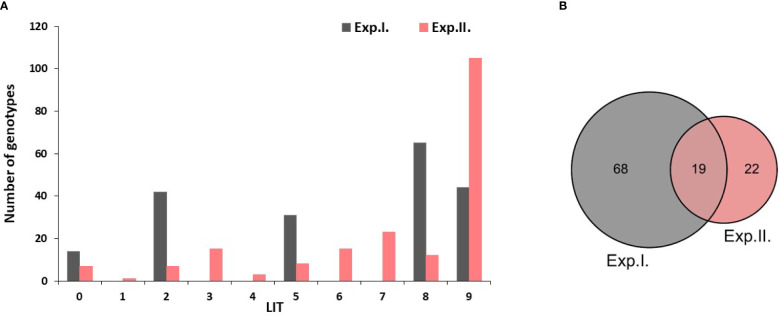
**(A)** Histogram represents the different genotypes' responses to PM infection in experiment I and II. Genotypes with a linear scale rangingfrom 0 to 5 are resistant.While genotypes with a score ranging from 6 to 9 are susceptible genotypes. **(B)** number of resistant genotypes in both experiments and the number of common resistant genotypes in both experiments.

The majority of the tested genotypes were found to be susceptible (LIT>5) to PM with a number of 109 and 155 genotypes in Exp. I. and Exp. II., respectively (**Figure 1A**). Few genotypes were found to be immune with a number of 14 and seven genotypes in Exp. I. and Exp. II., respectively. Moreover, a number of 73 and 34 genotypes showed degrees of resistance with 0<LIT ≤ 5 in Exp. I. and Exp. II., respectively. None of the tested genotypes was found to be immune in both experiments. However, 19 genotypes were found to be common resistant genotypes (0<LIT ≤ 5) in both experiments (**Figure 1B**). The list of these common resistant genotypes, their country of origin, and their reaction in each experiment are presented in **Table 2**, **S4**. Notably, these genotypes belonged to nine different countries.

**Table 2:** Summary of common resistant genotypes in Exp.I. and Exp.II, their country of origin, and their average IT in each experiment.

Genotype country	Number of genotypes	Average seedling reaction to powdery mildew	
		Exp.I.	Exp.II.
Canada	1	1	1.33
Egypt	7	1.25	1.17
Germany	1	2	1
Greece	3	1.75	1.25
Iran	1	2	1.67
Morocco	2	1	1.33
Oman	1	2	1.33
Tunisia	1	1	1.33
United Kingdom	1	1	1
Unknown	1	2	0

### 3.3 Association mapping of PM seedling resistance

#### 3.3.1 GWAS for PM seedling resistance in each experiment

Due to the presence of highly significant differences between the two experiments based on the ANOVA results, GWAS was conducted for each experiment separately. For each experiment, three different GWAS models were used for each genotypic data set separately. The tested population was reported to contain three different subpopulations (**Figure S1**) (Mourad et al., 2020). Therefore, for each model, PCA, Kin, or PCA+Kin were included to correct the effect of this population structure. A summary of all the identified significant markers for each experiment using each marker set is presented in **Table 3** while the detailed results are presented in **Table S5**.

**Table 3:** Summary of significant markers identified in each experiment based on genome-wide association study using 25K-SNP array and GBS-SNPs and the high confidence genes that they are located within.

Marker set	Total no. of sig markers	Experiment	No. of markers	No. of Chrom	R^2^	*P-*value	Allele effect	Number of genes		Best GWAS model	
Exp.I	52	25K-SNP array	37	11	0.82 – 7.31%	7.19E-06 – 0.001	(-1.66) – (-0.79)	25		FarmCPU+Kin+PCA	
		GBS-SNPs	15	6	4.68 – 18.27%	1.29E-05 – 0.001	(-2.02) – (-1.25)	5		FarmCPU+Kin	

**Figure 2:**
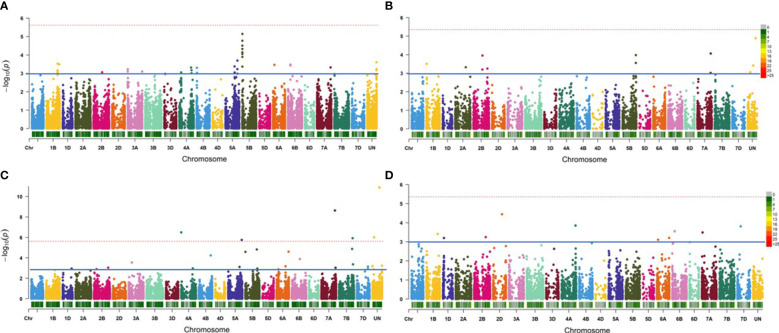
Manhattan plot represents the significant markers associated with powdery mildew resistance in the 198-spring wheat genotypes. **(A)** Significant 25K-SNP markers associated with the resistance in Exp.l. using FarmCPU+Kin+PCA model. **(B)** Significant GBS-SNP markers associated with the resistance in Exp.l. using FarmCPU+Kin.**(C)** Significant 25K-SNP associated with the resistance in Exp.II. using FarmCPU+Kin+PCA model. **(D)** Significant GBS-SNP markers associated with the resistance in Exp. II. using MLM+Kin model.

In Exp. I., the best GWAS model for the 25K-SNP array was FarmCPU+Kin+PCA, while the best model for the GBS-SNP was FarmCPU+Kin (**Figures S2A, B**). These two models identified a total of 52 significant markers in all with a number of 37 and 15 25K-SNP markers and GBS-SNPs, respectively (**Table 3** and **Figures 2A, B** **)**. All the significant markers identified in this experiment using the 25K-SNP set were found to have minor effects on PM seedling resistance and explained 0.82-7.31% of the phenotypic variations (R^2^). These markers were found to be distributed among 11 different chromosomes. For the GBS-SNPs, nine markers were found to have a major effect on the resistance while the remaining six markers had minor effects (**Table S5**).

In Exp. II., the best GWAS models were FarmCPU+PCA+Kin and MLM+kin for the 25K-SNPs array, respectively (**Figures S2C, D**). These two models identified 19 markers significantly associated with the resistance in this experiment with a number of 14 and five significant markers using 25K-SNPs and GBS-SNPs, respectively (**Figures 2C**, **D**). Most of the 25K-SNPs significant markers were found to have minor effects on the resistance with a number of ten minor and four major markers (**Table S5**). While all the GBS-SNPs significant markers were found to have minor effects on the resistance except one marker “S6A_58591217” on chromosome 6A which had a major effect with an R^2^ value of 13.42%.

**Figure 3:**
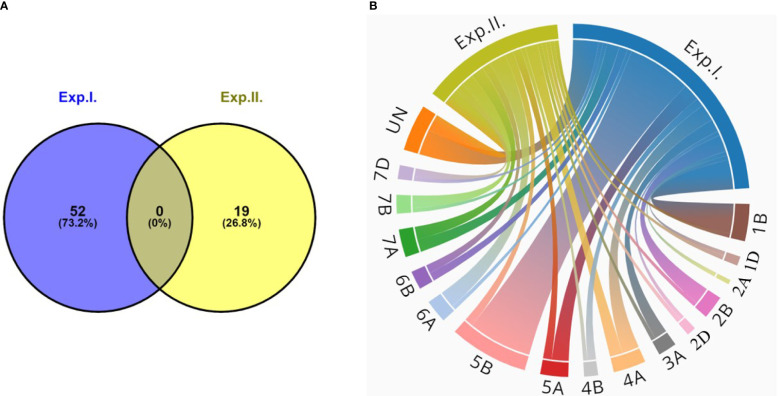
**(A)** Number of common significant markers between the two powdery mildew seedling experiments (Exp.l. and Exp.ll). **(B)** Chord diagram represents number of significant markers on each wheat chromosome associated with powdery mildew seedling resistance in both experiments (Exp.l. and Exp.ll.).

Combining the GWAS results of the two experiments, a total of 71 significant markers were identified to be significantly associated with the seedling resistance of the Egyptian isolates of *Bgt*. These markers were found to be distributed among 16 chromosomes of the wheat genome. None of these markers were found to be common between the two experiments (**Figure 3A**). Out of these 16 chromosomes, chromosome 5B was found to carry the highest number of significant markers with a total of 16 significant markers (**Figure 3B**).

#### 3.3.2 Gene models harboring the SNP markers associated with PM seedling resistance

To provide more understanding of the genetic control of PM seedling resistance in the studied materials, gene models harboring the significant markers in each experiment were investigated. In total, 28 and eight gene models were found to harbor the significant markers identified in Exp. I. and Exp. II., respectively (**Table 3**). The chromosomal position (bp) and the functional annotation of each gene model were presented in detail in **Table S5**. Furthermore, networks represent the relationship between the functional annotation of each gene and disease resistance is presented in **Figure S3**. Almost all the identified gene models in both experiments have a relationship with disease resistance in wheat such as stripe rust. Moreover, some gene models have a relationship with increasing plant immunity, innate immune response, controlling the response to biotic stimulus, and controlling the defense response.

In total 36 gene models were identified based on both evaluations. None of these gene models was found to commonly control the resistance in both experiments. Chromosome 5B was found to carry the highest number of significant markers and was found also to contain most of the gene models with a total of seven genes. Therefore, we will focus deeply on the genomic regions on this chromosome and give more concern to its role in controlling PM resistance in the current study.

### 3.4 Putative genomic regions on chromosome 5B

Chromosome 5B was found to carry 16 significant markers associated with the resistance in both experiments (**Table S5**). These markers were found to be located within seven different gene models. Therefore, more analyses were performed on these genomic regions.

#### 3.4.1 Haplotype block analysis of chromosome 5B

**Figure 4:**
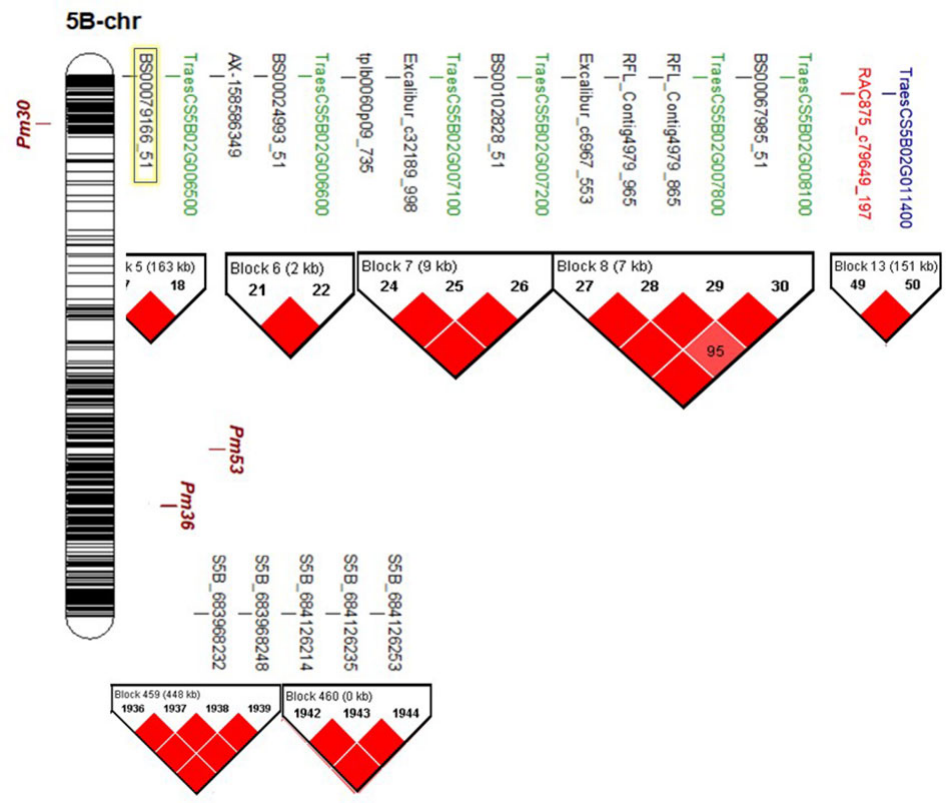
Chromosomal location (bp) of known powdery mildew resistant genes *(Pm30, Pm36* and *Pm53)* on chromosome 58, and the position of the identified significant markers on the same chromosome, gene models that they are located within, and haplotype block analysis. Highlighted SNP marker was reported previously to associated with fusarium head blight resistance in winter wheat (Aviles et al., 2020).

Haplotype block analysis detected the presence of 512 blocks on chromosome 5B (**Figure S4A**). The significant markers identified in the current study were distributed across seven blocks (blocks number 5, 6, 7, 8, 13, 459, and 460) (**Figure 4**, **S4B**). Remarkably, all the significant 25K-SNPs were located on the short arm of the chromosome and located within five blocks. While the significant GBS-SNPs were located on the long arm of the chromosome within two blocks. Block numbers 7 and 8 were found to contain seven significant markers located within three gene models. Three significant markers, BS00079166_51, BS00024993_51, and RAC875_c79649_197, were found to be located on blocks 5, 6, and 13.

#### 3.4.2 Gene enrichment analysis of gene models harboring significant markers on chromosome 5B

**Figure 5:**
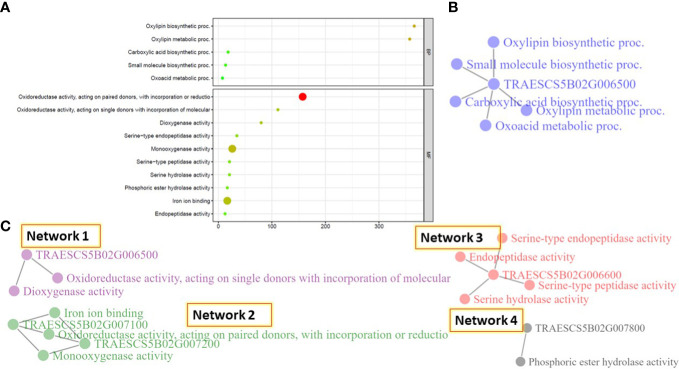
Gene enrichment analysis of the identified gene models on chromosome SB. **(A)** the identified pathways based on the biological process and cellular components, **(B)** gene network identified based on the biological process, and **(C)** the different networks identified based on the cellular components.

Gene enrichment for all the identified gene models located on 5B chr. was investigated. Using a cutoff 1% false discovery rate, five and ten pathways were identified based on the biological process (BP) and molecular function (MC) pathways (**Figure 5A** and **Table S6**). No significant pathways were identified for the studied gene models based on the cellular components.

The five BP pathways were found to be controlled by one gene model, *TRAESCS5B02G006500*, and working together in one network (**Figure 5B**). This network mainly controls the oxidative metabolism of polyunsaturated fatty acids. While the ten MF pathways performed four different networks (**Figure 5C**). Network 1 was found to mainly control the catalysis of an oxidation-reduction (redox) in the plant cell and was controlled by *TRAESCS5B02G006500* gene model. The same function was found to be controlled by Network 2, however, it was controlled by two different gene models, *TRAESCS5B02G007100* and *TRAESCS5B02G007200*, that seem to work separately from *TRAESCS5B02G006500* gene model. Network 3 was found to be controlled by one gene model, *TRAESCS5B02G006600*, and mainly controlled the catalysis reaction of polypeptide chain elongation. Network 4, controlled by *TRAESCS5B02G007800* gene model, was found to mainly control the hydrolysis of any phosphoric ester bond.

#### 3.4.3 Gene network of the seven identified gene models harboring significant markers on chromosome 5B

**Figure 6:**
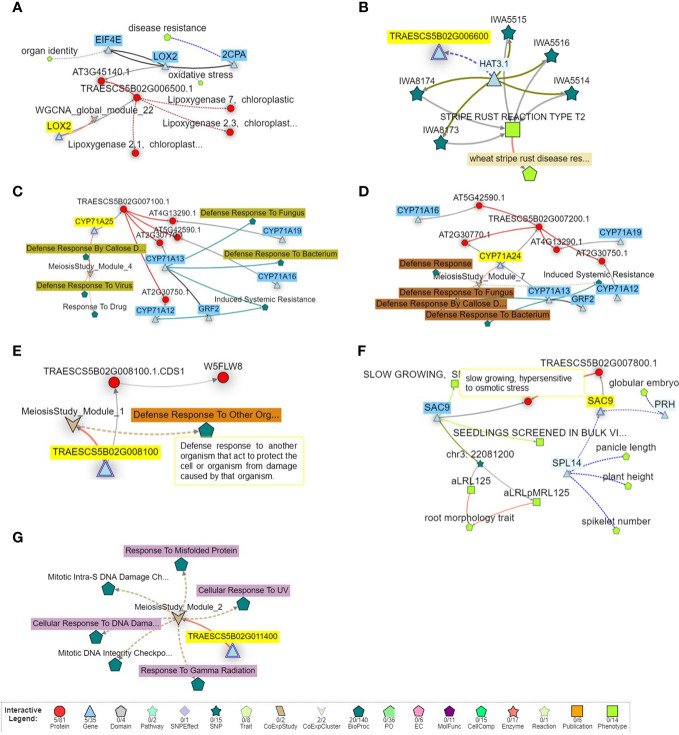
Gene network of the seven gene models identified on chromosome SB harbors significant markers associated with powdery mildew seedling resistance. **(A)** network of *TraesCS5802G006500* gene model. **(B)** network of *TraesCS5B02G006600* gene. **(C)** network of *TraesCS5802G007100* gene. **(D)** network of *TraesCS5802G007200* gene model. **(E)** network of *TraesCS5802G008100* gene model. **(F)** network of *TraesCS5802G007800* gene model **(G)** network of *TraesCS5802G011400* gene model.

To provide more information on the identified putative genomic region, the function of the identified gene models harboring this region with disease resistance was investigated and presented in **Figure 6**. Remarkably, two gene models were directly associated with disease resistance in the plant such as *TraesCS5B02G006500* (known as *2CPA* gene) that controls disease resistance in wheat, and *TraesCS5B02G006600* (known as *HAT3.1* gene) that controls wheat stripe rust resistance (**Figures 6A, B** **)**. Three other gene models, *TraesCS5B02G007100*, *TraesCS5B02G007200*, and *TraesCS5B02G008100*, were found to control wheat defense against bacteria, fungus, viruses, and other organisms, as well as induce the systematic resistance in wheat (**Figures 6C–E**). No direct relation between disease resistance and the remaining two gene models was found. However, *TraesCS5B02G007800* was found to control the slow growth of wheat plants in response to osmotic stress, a response that produces a hypersensitive reaction to the stress (**Figure 6F**). Moreover, *TraesCS5B02G011400* was found to control the response of wheat cells to DNA damage in response to environmental insults or any error occurring during metabolism (**Figure 6G**).

### 3.5 Selection of the most promising superior resistant genotypes

**Figure 7:**
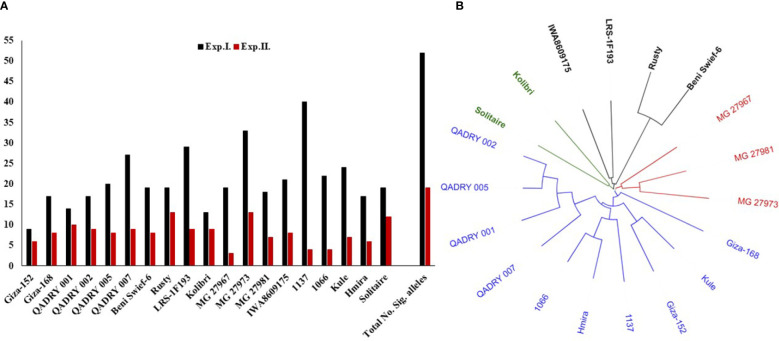
**(A)** Number of target alleles in each of the 19 common resistant genotypes in both evaluations (Exp. (I) and Exp.II.). **(B)** The genetic distance between each pair of these common resistant genotypes.

The total number of target alleles of significant markers was investigated in the resistant genotypes (**Figure 7**). Out of the 52 significant markers associated with the resistance in Exp. I., 40 alleles were found in the genotype “1137” from Morocco. While the lowest number of the target alleles was found in the Egyptian genotype “Giza152”. The highest number of target alleles associated with the resistance in Exp. II. was found in “MG27973” and “Rusty” genotypes which contain 13 alleles out of the 19 significant alleles identified in Exp. II. On the other hand, the “MG27967” genotype contained the lowest number of significant alleles associated with the resistance in this experiment. Combining the number of significant alleles associated with the resistance in both experiments, “MG27973” genotypes from Greece contained the highest number of target alleles followed by “1137” with a number of 46 and 44 alleles, respectively. The number of the target alleles in the Egyptian genotypes ranged from 15 alleles in “Giza152” to 36 alleles in “QADRY007” breeding line.

The genetic distance among the 19 common resistant genotypes was calculated and presents in **Figure 7B** and **Table S7**. Based on the genetic distance, the 19 genotypes were clustered in four different clusters. The three Greek genotypes, including “MG27973” genotype, were clustered together in one group. Furthermore, “MG27973” genotype had a high genetic distance with values ranging from 0.3509 between it and “MG27967” to 0.4792 between it and the Egyptian genotype BeniSwief-6 (**Table S7**). The Moroccan genotype “1137” was clustered in the same group as most of the Egyptian genotypes **Figure 7B**.

## 4 Discussion

Wheat powdery mildew (PM) caused by *Blumeria graminis* f.sp. *tritici* is a serious disease that causes huge damage to wheat yield. Most Egyptian bread wheat cultivars were reported to be moderately resistant or susceptible to PM (El-Shamy et al., 2016; Abdelrhim et al., 2018; Draz et al., 2019; El-Shamy and Mohamed, 2022). Therefore, looking for new sources of resistance is urgently needed. In the current study, a highly diverse wheat panel collected from 22 different countries around the world was evaluated for their resistance to the recent *Bgt* Egyptian populations. This panel was reported to be genetically highly diverse and adapted to Egyptian field conditions (Mourad et al., 2020; Abou-Zeid and Mourad, 2021; Hasseb et al., 2022; Mourad et al., 2022a; Mourad et al., 2022b). Such highly diverse plant materials will be very helpful in detecting the possible genetic resources for improving PM resistance in Egyptian wheat. The evaluation was done in two different experiments (Exp. I. and Exp. II.) using two isolate populations (*Bgt1* and *Bgt2*) that were collected from the Egyptian fields in the last two growing seasons (2020 and 2021). These populations were reported to contain different isolates (Dr. Samar Esmail, personal communications). Recent studies reported the existence of different *Bgt* isolate in each growing season in the Egyptian fields (El-Shamy and Mohamed, 2022). Therefore, diverse *Bgt* isolates were supposed to be used in the current evaluations. The susceptible check “Jagalene” had a high susceptible IT (IT=4) on both evaluations which confirms the efficiency of the artificial inoculation in both experiments.

### 4.1 Effective PM resistance genes against Bgt Egyptian isolate

The evaluation of the 20 isolines was done in each experiment separately to investigate the changes in the genetic control of PM resistance under Egyptian conditions. Such changes in the resistance shed light on the changes that occurred in *Bgt* isolates during the different growing seasons. The eight immune/resistant isolines were reported previously to be resistant under Egyptian conditions (El-Shamy et al., 2016; Abdelrhim et al., 2018; El-Shamy and Mohamed, 2022). Some isolines showed different resistant responses in the two experiments confirming the presence of different *Bgt* isolates in each evaluation. Interestingly, *Pm35*, *Pm3a*, *Pm4b*, were found to be effective against the *Bgt* isolates used in Exp.I. and ineffective in Exp. II. These genes were reported as effective genes against the Egyptian *Bgt* isolates collected from different fields including Kafrelsheikh (Abdelrhim et al., 2018). The changes in the efficiency of these isolines in Exp. II. confirming the appearance of highly aggressive *Bgt* isolates that increase the need for improving PM resistance in the Egyptian wheat germplasm.

### 4.2 Genetic variation in PM seedling resistance in the studied spring wheat panel

The presence of highly significant differences between the two experiments concluded the significant differences between the two populations of *Bgt* isolates used in this study (**Table 1**). Furthermore, the presence of highly significant differences among the tested genotypes in each experiment confirmed the high genetic variation in the tested panel. Thus, the selection of highly resistant genotypes could be done using the currently evaluated panel. The high degree of broad-sense heritability in both experiments indicated that the phenotypic variation observed in each experiment is mainly based on genetic variation. Therefore, the selected genotypes based on the current evaluations will be promising due to their stable response. High degrees of heritability for PM seedling resistance were reported previously (Hoseinzadeh et al., 2019; Hinterberger et al., 2022). Such high degrees of heritability concluded the promising genetic improvement for PM seedling resistance using the current panel.

Notably, most of the tested genotypes were found to be susceptible to PM suggesting that improving PM resistance in the bread wheat germplasm under the Egyptian conditions is required. Moreover, most of the Egyptian genotypes were found to be moderately resistant or susceptible. The absence of immune Egyptian wheat genotypes was reported previously in most of the commercial cultivars (Emara et al., 2016; Draz et al., 2019; Elsayed and Elkot, 2020; Draz et al., 2022). The 19 common resistant genotypes in both experiments were found to be moderately resistant to the studied *Bgt* isolates. All these common genotypes had almost similar resistance responses to the Egyptian genotypes. However, crossing among these genotypes could help obtain broad-spectrum resistance against different Egyptian *Bgt* isolates as they belong to different genetic backgrounds.

### 4.3 Association mapping of PM seedling resistance

Unlike rust diseases in Egyptian wheat, little concern was drawn into the genetic control of PM resistance under Egyptian conditions. Some previous studies tested the presence of specific PM resistance genes in the Egyptian wheat germplasm without knocking into the new sources of genetic control (Emara et al., 2016; Elsayed and Elkot, 2020; Draz et al., 2022). In this study, the association mapping of PM resistance was studied for the first time under Egyptian conditions. Two types of genetic markers (GBS-SNPs and 25K i-select SNP array) were used in conducting GWAS analysis. Both marker sets were reported to be effective and cover different parts of the wheat genome (Liu et al., 2020; Mourad et al., 2022b; Esmail et al., 2023). The GWAS models used in the current study were reported to be effective in correcting the effect of population structure and detecting the significant markers (Turuspekov et al., 2016; Kaler et al., 2020; Muhammad et al., 2020, Muhammad et al., 2021; Mourad et al., 2022b; Esmail et al., 2023). The best model differs based on the phenotypic evaluation and the marker set. Therefore, testing the three GWAS models in each study is recommended. A similar conclusion was obtained in a previous study (Mourad et al., 2022a). The results of this study shed light on genomic regions controlling PM resistance under Egyptian conditions.

Different significant markers were found to control the resistance in each experiment. This further supports that the genotypes were exposed to different *Bgt* races in the two growing seasons due to the fungus’s ability to mutate and produce new pathotypes. Hence, more effort should be done to improve PM resistance in the Egyptian fields. Combining the GWAS results of both experiments, 71 MTAs across 16 different chromosomes were detected. The presence of many MTAs controlling PM resistance across different chromosomes was reported previously in different wheat backgrounds such as American wheat, Russian wheat, winter wheat, and other backgrounds (Liu et al., 2017; Kang et al., 2019; Leonova, 2019; Alemu et al., 2021; Du et al., 2021). Most of the identified significant markers were located within gene models that functionally annotated to directly or indirectly control wheat resistance to different organisms. Moreover, “Kukri_c6266_260” marker on chromosome 5A was reported to control PM in many regions in Europe such as Lithuania, Sweden, and Denmark (Alemu et al., 2021). In our study, this marker was found to minorly control the resistance in Exp. I. It was located within *TraesCS5A02G421600* gene that was annotated to produce S-Adenosyl-l-methionine methyltransferase (SAMT) protein. This protein was reported to be involved in the plant defense process (Ross et al., 1999). Based on our findings and the previous findings, we conclude that this marker could provide broad-spectrum resistance against many *Bgt* pathotypes. The marker-trait association identified in this study is accurate and precise.

### 4.4 Insights into the putative genomic regions on chromosome 5B

The majority of the significant markers identified in this study are located on chromosome 5B. Previous studies concluded the presence of QTLs controlling fungal disease resistance in wheat on this chromosome (Salina et al., 2018). Moreover, some of the significant markers detected in the recent study were reported to be associated with disease resistance in wheat such as; “BS00079166_51” which was previously reported to be associated with fusarium head blight (FHB) resistance in winter wheat (Aviles et al., 2020), and “tplb0060p09_735”, “BS00024993_51”, and “BS00102828_51” markers that were developed for KASP markers for *Lr52* and *Yr47* rust genes (Qureshi et al., 2017). These four validated SNP markers could be used in MAS for resistance to various wheat diseases after further validation in a different genetic background (Sallam et al., 2023). More understanding of the role of these regions in PM resistance is required.

All the 5B-significant markers were located in seven blocks based on the haplotype block analysis. Significant markers located in the same block are most likely controlling the same QTL. The significant marker identified in Exp.II. was located separately in block 13 and far from the blocks controlling the resistance in Exp.I. This confirms that the genetic control of PM in both experiments is different due to the presence of different *Bgt* populations. It was reported that selecting blocks that are rich in significant markers are better than selecting individual SNPs in MAS (Mokry et al., 2014; Mourad et al., 2018). Therefore, the recently identified blocks could be used in MAS for PM seedling resistance. The identified significant markers and their harboring blocks were found to contain a total of seven genes all located on the short arm of the chromosome. All these genes had a relationship with disease resistance in wheat which confirms our results. Gene enrichment decreased the number of these genes to five genes that significantly control biological processes and/or molecular function pathways in the wheat genome. All the pathways identified based on the biological processes were found in one network and controlled by *TraesCS5B02G006500* gene. These pathways mainly control the metabolism of some important hormones that are involved in plant defense. For example, oxylipin hormone was reported as a major hormone produced by jasmonic acid (JA) that is very important in controlling the plant defense system (Wang et al., 2021; Esmail et al., 2023). Furthermore, carboxylic acid and oxylipin hormone was found as a major component of the glycerol-induced powdery mildew resistance in wheat (Li et al., 2020). Recent studies reported that oxoacid metabolic processes and carboxylic acid pathways are involved in the resistance of wheat genotypes to leaf rust (Dorostkar et al., 2022). Furthermore, the same gene model was found to control two molecular function pathways that are important in the catalysis of an oxidation-reduction (redox) reaction in wheat. The regulation of redox changes in plant cells is a preliminary response to the infection that includes the hypersensitive response in the resistance genotypes (Matika and Loake, 2014). Three other networks were identified based on the molecular function process that was also involved in disease resistance/susceptibility in the plant. For example, network 2 controlling the activity of monooxygenase and iron binding processes was reported to negatively regulate the resistance of wheat diseases such as fusarium head blight (Herlihy et al., 2020; Ma et al., 2022). Network 3, controlled by *TraesCS5B02G006600* gene model, was found to contain four different pathways that control the activity of serine hydrolase in wheat. The regulation of serine/threonine was involved in wheat resistance to Septoria blotch disease (Benbow et al., 2020; Zhou et al., 2020). Furthermore, Serine/threonine kinase gene was reported as a key member of *Pm21* powdery mildew resistance gene (Cao et al., 2011). Network 4 controls the phosphoric ester hydrolase activity, a process that involved in the plant-pathogen interaction during wheat stem rust infection (Kataria and Kaundal, 2022). The presence of different networks based on the molecular functions pathways suggests that the identified genomic region contains more than one gene. All the genes present in the identified genomic regions on the short arm of 5B have a significant role in fungal disease resistance in wheat.

To provide more understanding of these gene models and PM resistance, we figured out the known *Pm* genes that were located on chromosome 5B. Five genes were previously mapped on this chromosome; *Pm30* (Liu et al., 2002), *Pm36* (Blanco et al., 2008), *Pm53* (Petersen et al., 2015), *PmAS846* (Xue et al., 2012), and *Pm16* (Chen et al., 2005). Out of these five genes, two were located on the short arm of the chromosome (*Pm30* and *Pm16*). While the remaining three genes were located on the long arm of the chromosome. Recent studies linked *Pm30* with three 90K-SNPs markers namely “wsnp_Ex_c13496_21243167”, “Kukri_c18702_132”, and “wsnp_Ex_c20988_30107609” (Liu et al., 2017). Comparing the chromosomal position of these three SNPs and the position of the significant markers identified in the current study, *Pm30* was located far from the genomic region identified on the short arm of the chromosome (**Figure 4**). Furthermore, one of the three mentioned markers in Liu et al. (2017)’study was included in our 25K-SNP array marker data and was not significantly associated with the resistance in both experiments (**Figure S5**). Unfortunately, there is no available SNP marker for *Pm16* gene. Therefore, it was hard to compare its position with the identified genomic region. However, *Pm16* was identified as an effective gene that controls PM resistance under the Egyptian conditions (El-Shamy et al., 2016). Due to the lack in the seed of *Pm16* isoline, we could not identify its reaction to the studied *Bgt* populations. However, due to the presence of seven gene models on the short arm of the chromosome, we can conclude that *Pm16* could be one of these genes.

Moreover, the chromosomal location of *Pm53* and *Pm36* genes located on the long arm of the chromosome was investigated. *Pm53* was reported to be flanked by IWA2698 and IWA6024 SNP markers (Petersen et al., 2015). Based on the *EnsemblePlants* database, these markers are located between 478,305,518bp and 478,305,718bp and are located within *TraesCS5B02G292800* gene model. Based on this location, *Pm53* is far from the genomic regions identified in our recent study (**Figure 4**). Moreover, in our marker data sets five SNP markers were found to have the same chromosomal location of *Pm53*. All these markers were located in block 194 and were not significantly associated with the resistance which confirms that the identified genomic region is different from *Pm53* gene. Following the same method, *Pm36* gene was mapped near to “IWB22904” SNP marker that was located within 540,266,445 and 541,343,146bp (Nigro et al., 2022). The chromosomal location of this gene was far from the significant marker detected in our study (**Figure 4**). Furthermore, we found four SNP markers in our marker data set that were located in the same position. Based on the haplotype block analysis, these markers were located in block 261 and block 262 and were not significantly associated with PM resistance (**Figure S5**). Furthermore, *Pm36* and *Pm53* isolines were found to be resistant in at least one experiment (**Table S2**). Based on the chromosomal location of *Pm36* and *Pm53*, it seems that the identified genomic region is novel and differs from these two genes.

Undoubtedly, more studies are required to provide more understanding of these putative genomic regions in controlling PM seedling resistance. However, based on our preliminary study, we can conclude that these regions seem to contain more than one PM resistance gene. *Pm16* could be one of these genes in addition to other unknown genes. Moreover, other disease-resistance genes such as FHB and rusts might contribute to PM resistance. Therefore, genes located in the identified genomic regions could have pleiotropic effects against many wheat diseases.

### 4.5 Selection of genotypes with broad-spectrum resistance to Egyptian Bgt populations

Gene pyramiding is preferred to produce genotypes with broad-spectrum resistance to PM. It requires crossing between resistant genotypes that carry different genes/MTAs controlling the resistance. Despite the approximately similar response of the 19 common genotypes, different numbers of target alleles of the significant markers were found in each one of these genotypes. Therefore, gene pyramiding could be possible using the current common genotype. The Grecian genotype “MG27973” and the Moroccan genotype “1137” contained a higher number of target alleles than all the Egyptian genotypes. Furthermore, the Egyptian breeding line Qadry_007 contains a higher number of targeted alleles among Egyptian genotypes. Crossing Qadry_007 with both the Moroccan and Grecian genotypes seems to be helpful in gene pyramiding for PM resistance. However, “MG27973” was found to be genetically distinct from all the Egyptian genotypes. On the other hand, “1137” was found to be located in the same group as most of the Egyptian genotypes. It was concluded that the best parents to be crossed to improve specific traits are those that are highly genetically distant (Bertan et al., 2007; Mourad et al., 2020; Mourad et al., 2022a). Therefore, using “MG27973” as a parent in future breeding programs for PM resistance will accelerate the Egyptian wheat germplasm. It is highly recommended to combine phenotypic selection with genetic analysis to identify the most promising genotypes (Eltaher et al., 2018). Using such a combined selection method will avoid human errors that could lead to misleading in the phenotypic selection process (Sallam et al., 2019). Many previous studies used this approach in selecting promising genotypes for different abiotic and biotic stresses (Abou-Zeid and Mourad, 2021; Eltaher et al., 2021; Mourad et al., 2021a; Abo-elyousr et al., 2022; Eltaher et al., 2022; Mourad et al., 2022b).

## 5 Conclusion

In conclusion, the high variation in the tested wheat panel suggests the possibility of selecting broad-spectrum resistant genotypes. A low number of immune genotypes were found confirming that more concern should be given to improving PM resistance under Egyptian conditions. This study is the first AM study that unlocked the genetic control of such a serious disease in the Egyptian fields. Novel genomic regions were found on chromosome 5B that differ from the known *Pm* genes mapped on this chromosome, except *Pm16.* The identified genomic regions were found to contain five different gene models, forming four molecular function networks, and located in five different haplotype blocks. Therefore, these genomic regions seem to contain more than one resistant gene. Some other disease-resistance genes were found in the same region suggesting that pleiotropic effects genes could be present in these regions. The Grecian selected genotype provides a good source for PM broad-spectrum resistance due to the high distance between it and Egyptian genotypes and its resistance to the different isolates of *Bgt*.

## Data availability statement

The original contributions presented in the study are included in the article/**Supplementary material**. Further inquiries can be directed to the corresponding author.

## Author contributions

AM designed the experiment, conducted all the genetic analyses, and drafted the manuscript. RH reviewed the manuscript. SE performed the phenotyping for powdery mildew resistance and helped in drafting the manuscript. All authors contributed to the article and approved the submitted version.

## References

[B1] Abdelrhim A. Abd-Alla H. M. Abdou E. S. Ismail M. E. Cowger C. (2018). Virulence of egyptian blumeria graminis f. sp. tritici population and response of egyptian wheat cultivars. Plant Dis. 102, 391–397. doi: 10.1094/PDIS-07-17-0975-RE 30673514

[B2] Abo-elyousr K. A. M. Mourad A. M. I. Baenziger P. S. Shehata A. H. A. Eckstein P. E. Beattie A. D.. (2022). Identification of putative SNP markers associated with resistance to Egyptian loose smut race (s) in spring barley. Genes (Basel) 13, 1–15. doi: 10.3390/genes13061075 PMC9223236 35741837

[B3] Abou-Zeid M. A. Mourad A. M. I. (2021). Genomic regions associated with stripe rust resistance against the Egyptian race revealed by genome-wide association study. BMC Plant Biol. 21, 1–14. doi: 10.1186/s12870-020-02813-6 33446120 PMC7809828

[B4] Aleksandrov V. Kartseva T. Alqudah A. M. Kocheva K. Tasheva K. Börner A.. (2021). Genetic diversity, linkage disequilibrium and population structure of bulgarian bread wheat assessed by genome-wide distributed SNP markers: From old germplasm to semi-dwarf cultivars. Plants 10, 1–20. doi: 10.3390/plants10061116 PMC8228972 34073128

[B5] Alemu A. Brazauskas G. Gaikpa D. S. Henriksson T. Islamov B. Jørgensen L. N.. (2021). Genome-wide association analysis and genomic prediction for adult-plant resistance to septoria tritici blotch and powdery mildew in winter wheat. Front. Genet. 12. doi: 10.3389/fgene.2021.661742 PMC8149967 34054924

[B6] Alqudaha A. M. Sallamb A. Baenzigerc P. S. Börnera A. (2019). GWAS: Fast-Forwarding Gene Identification in Temperate Cereals: Barley as a Case Study-A review. J. Adv. Res. 4:119–135. doi: 10.1016/j.jare.2019.10.013 PMC6961222 31956447

[B7] Amro A. Harb S. Youssef K. Ali M. M. F. Mohammed A. G. Mourad A. M. I.. (2022). Growth responses and genetic variation among highly ecologically diverse spring wheat genotypes grown under seawater stress. Front. Plant Sci. 13. doi: 10.3389/fpls.2022.996538 PMC9614663 36311097

[B8] Aviles A. C. Harrison S. A. Arceneaux K. J. Brown-Guidera G. Mason R. E. Baisakh N. (2020). Identification of qtls for resistance to fusarium head blight using a doubled haploid population derived from southeastern united states soft red winter wheat varieties ags 2060 and ags 2035. Genes (Basel). 11, 1–18. doi: 10.3390/genes11060699 PMC7349885 32630440

[B9] Barrett J. C. Fry B. Maller J. Daly M. J. (2005). Haploview: analysis and visualization of LD and haplotype maps. Bioinformatics 21, 263–265. doi: 10.1093/bioinformatics/bth457 15297300

[B10] Bates D. Mächler M. Bolker B. Walker S. (2015). Fitting linear mixed-effects models using lme4. J. Stat. Software 67, 1–48. doi: 10.18637/JSS.V067.I01

[B11] Benbow H. R. Brennan C. J. Zhou B. Christodoulou T. Berry S. Uauy C.. (2020). Insights into the resistance of a synthetically-derived wheat to septoria tritici blotch disease: Less is more. BMC Plant Biol. 20, 1–23. doi: 10.1186/s12870-020-02612-z 32883202 PMC7469286

[B12] Bertan I. De Carvalho F. I. F. De Oliveira A. C. (2007). Parental selection strategies in plant breeding programs. J. Crop Sci. Biotechnol. 10, 211–222. Available at: https://koreascience.kr/article/JAKO200716637994242.page.

[B13] Blanco A. Gadaleta A. Cenci A. Carluccio A. V. Abdelbacki A. M. M. Simeone R. (2008). Molecular mapping of the novel powdery mildew resistance gene Pm36 introgressed from triticum turgidum var. dicoccoides in durum wheat. Theor. Appl. Genet. 117, 135–142. doi: 10.1007/s00122-008-0760-0 18392800

[B14] Bradbury P. J. Zhang Z. Kroon D. E. Casstevens T. M. Ramdoss Y. Buckler E. S. (2007). TASSEL: Software for association mapping of complex traits in diverse samples. Bioinformatics 23, 2633–2635. doi: 10.1093/bioinformatics/btm308 17586829

[B15] Browder L. E. (1971). Pathogenic specialization in cereal rust fungi, especially Puccinia recondita I.sp. tritici: Concepts, methods of study and application ,” in Concepts, methods of study and application (U.S. Dep. Agric. Tech), 1432.

[B16] Cao A. Xing L. Wang X. Yang X. Wang W. Sun Y.. (2011). Serine/threonine kinase gene stpk-V, a key member of powdery mildew resistance gene Pm21, confers powdery mildew resistance in wheat. Proc. Natl. Acad. Sci. USA 108, 7727–7732. doi: 10.1073/pnas.1016981108 21508323 PMC3093467

[B17] Chen X. M. Luo Y. H. Xia X. C. Xia L. Q. Chen X. Ren Z. L.. (2005). Chromosomal location of powdery mildew resistance gene Pm16 in wheat using SSR marker analysis. Plant Breed. 124, 225–228. doi: 10.1111/j.1439-0523.2005.01094.x

[B18] Cheng P. Guo M. Hao X. Guo X. Yao Q. Guo Q.. (2022). Evaluation of powdery mildew resistance and molecular detection of resistance genes in an international wheat collection. Crop Prot. 160, 0261–2194. doi: 10.1016/j.cropro.2022.106033

[B19] Costamilan L. M. (2005). Variability of the wheat powdery mildew pathogen blumeria graminis f. sp. tritici. Fitopatol. Bras. 30, 420–422. doi: 10.1590/s0100-41582005000400015

[B20] Daba S. D. Tyagi P. Brown-guedira G. Mohammadi M. (2018). Genome-wide association studies to identify loci and candidate genes controlling kernel weight and length in a historical united states wheat population. Front. Plant Sci. 9. doi: 10.3389/fpls.2018.01045 PMC6086202 30123226

[B21] Dorostkar S. Dadkhodaie A. Ebrahimie E. Heidari B. Ahmadi-Kordshooli M. (2022). Comparative transcriptome analysis of two contrasting resistant and susceptible aegilops tauschii accessions to wheat leaf rust (Puccinia triticina) using RNA-sequencing. Sci. Rep. 12, 1–19. doi: 10.1038/s41598-021-04329-x 35039525 PMC8764039

[B22] Draz I. S. Elkot A. F. Abdelrhim A. S. (2022). Allelism and resistance loci of powdery mildew and leaf rust in Egyptian hexaploid bread wheat. Cereal Res. Commun. 50, 85–93. doi: 10.1007/s42976-021-00163-z

[B23] Draz I. S. Esmail S. M. Abou-Zeid M. A. E. H. Essa T. A. E. M. (2019). Powdery mildew susceptibility of spring wheat cultivars as a major constraint on grain yield. Ann. Agric. Sci. 64, 39–45. doi: 10.1016/j.aoas.2019.05.007

[B24] Du X. Xu W. Peng C. Li C. Zhang Y. Hu L. (2021). Identification and validation of a novel locus, qpm-3BL, for adult plant resistance to powdery mildew in wheat using multilocus GWAS. BMC Plant Biol. 21, 1–13. doi: 10.1186/s12870-021-03093-4 34330216 PMC8323325

[B25] Elsayed M. Elkot A. (2020). Molecular identification of some powdery mildew resistance genes in ten Egyptian durum wheat cultivars. J. Plant Prot. Pathol. 11, 205–209. doi: 10.21608/jppp.2020.87592

[B26] El-Shamy M. M. Emara H. M. Mohamed M. E. (2016). Virulence analysis of wheat powdery mildew (Blumeria graminis f. sp. tritici) and effective genes in middle delta, Egypt. Plant Dis. 100, 1927–1930. doi: 10.1094/PDIS-01-16-0130-RE 30682990

[B27] El-Shamy M. M. Mohamed M. E. (2022). Virulence analysis of wheat powdery mildew races during 2019–2020 seasons in Egypt. Cereal Res. Commun. 50, 67–73. doi: 10.1007/s42976-021-00156-y

[B28] El-Shamy M. Sallam M. Awad H. (2012). Powdery mildew infection on some Egyptian bread wheat cultivars in relation to environmental conditions. J. Plant Prot. Pathol. 3, 363–372. doi: 10.21608/jppp.2012.83777

[B29] Eltaher S. Mourad A. M. I. Baenziger P. S. Wegulo S. Belamkar V. Sallam A. (2021). Identification and validation of high LD hotspot genomic regions harboring stem rust resistant genes on 1B, 2A (Sr38), and 7B chromosomes in wheat. Front. Genet. 12. doi: 10.3389/fgene.2021.749675 PMC8517078 34659366

[B30] Eltaher S. Sallam A. Belamkar V. Emara H. A. Nower A. A. Salem K. F. M.. (2018). Genetic diversity and population structure of F3:6 Nebraska winter wheat genotypes using genotyping-by-sequencing. Front. Genet. 9. doi: 10.3389/fgene.2018.00076 PMC5857551 29593779

[B31] Eltaher S. Sallam A. Emara H. A. Nower A. A. Salem K. F. M. Börner A.. (2022). Genome-wide association mapping revealed SNP alleles associated with spike traits in wheat. Agronomy 12, 1–19. doi: 10.3390/agronomy12061469

[B32] Emara H. M. Omar A. F. El-Shamy M. M. Mohamed M. E. Emara H. M. Omar A. F.. (2016). Identification of Pm24, Pm35 and Pm37 in thirteen Egyptian bread wheat cultivars using SSR markers. Ciec. e Agrotecnol. 40, 279–287. doi: 10.1590/1413-70542016403036315

[B33] Esmail S. M. Omar G. E. Mourad A. (2023). In-depth understanding of the genetic control of stripe rust resistance (Puccinia striiformis f. sp. tritici) induced in wheat (Triticum aestivum l.) by trichoderma asperellum T34. Plant Dis. 107, 457–472. doi: 10.1094/PDIS-07-22-1593-RE 36449539

[B34] Figureueroa M. Hammond-Kosack K. E. Solomon P. S. (2018). A review of wheat diseases–a field perspective. Mol. Plant Pathol. 19, 1523–1536. doi: 10.1111/mpp.12618 29045052 PMC6638159

[B35] Ge S. X. Jung D. (2018). ShinyGO: a graphical enrichment tool for ani-mals and plants. bioRxiv 36, 315150. doi: 10.1101/082511.Lai PMC7178415 31882993

[B36] Hassani-Pak K. Singh A. Brandizi M. Hearnshaw J. Parsons J. D. Amberkar S.. (2021). KnetMiner: a comprehensive approach for supporting evidence-based gene discovery and complex trait analysis across species. Plant Biotechnol. J. 19, 1670–1678. doi: 10.1111/pbi.13583 33750020 PMC8384599

[B37] Hasseb N. M. Sallam A. Karam M. A. Gao L. Wang R. R. C. Moursi Y. S. (2022). High-LD SNP markers exhibiting pleiotropic effects on salt tolerance at germination and seedlings stages in spring wheat. Plant Mol. Biol. 108, 585–603. doi: 10.1007/s11103-022-01248-x 35217965 PMC8967789

[B38] He H. Liu R. Ma P. Du H. Zhang H. Wu Q.. (2021). Characterization of Pm68, a new powdery mildew resistance gene on chromosome 2BS of Greek durum wheat TRI 1796. Theor. Appl. Genet. 134, 53–62. doi: 10.1007/s00122-020-03681-2 32915283

[B39] Herlihy J. H. Long T. A. McDowell J. M. (2020). Iron homeostasis and plant immune responses: Recent insights and translational implications. J. Biol. Chem. 295, 13444–13457. doi: 10.1074/jbc.REV120.010856 32732287 PMC7521657

[B40] Hinterberger V. Douchkov D. Lück S. Kale S. Mascher M. Stein N.. (2022). Mining for new sources of resistance to powdery mildew in genetic resources of winter wheat. Front. Plant Sci. 13. doi: 10.3389/fpls.2022.836723 PMC8922026 35300015

[B41] Hoseinzadeh P. Zhou R. Mascher M. Himmelbach A. Niks R. E. Schweizer P.. (2019). High resolution genetic and physical mapping of a major powdery mildew resistance locus in barley. Front. Plant Sci. 10. doi: 10.3389/fpls.2019.00146 PMC6382739 30838011

[B42] Huang L. S. Zhang D. Y. Liang D. Yuan L. Zhao J. L. Hu G. S.. (2013). Continuous wavelet analysis for diagnosing stress characteristics of leaf powdery mildew. Int. J. Agric. Biol. 15, 34–40. doi: 12–192/2013/15–1–34–40

[B43] Hurni S. Brunner S. Stirnweis D. Herren G. Peditto D. McIntosh R. A.. (2014). The powdery mildew resistance gene Pm8 derived from rye is suppressed by its wheat ortholog Pm3. Plant J. 79, 904–913. doi: 10.1111/tpj.12593 24942074

[B44] Kaler A. S. Gillman J. D. Beissinger T. Purcell L. C. (2020). Comparing different statistical models and multiple testing corrections for association mapping in soybean and maize. Front. Plant Sci. 10. doi: 10.3389/fpls.2019.01794 PMC7052329 32158452

[B45] Kang Y. Barry K. Cao F. Zhou M. (2019). Genome-wide association mapping for adult resistance to powdery mildew in common wheat. Mol. Biol. Rep. 47, 1241–1256. doi: 10.1007/s11033-019-05225-4 31813131

[B46] Kataria R. Kaundal R. (2022). Deciphering the crosstalk mechanisms of wheat-stem rust pathosystem: Genome-scale prediction unravels novel host targets. Front. Plant Sci. 13. doi: 10.3389/fpls.2022.895480 PMC9253690 35800602

[B47] Kersey P. J. Allen J. E. Armean I. Boddu S. Bolt B. J. Carvalho-Silva D.. (2016). Ensembl genomes 2016: More genomes, more complexity. Nucleic Acids Res. 44, D574–D580. doi: 10.1093/nar/gkv1209 26578574 PMC4702859

[B48] Kumar D. Kumar A. Chhokar V. Gangwar O. P. Bhardwaj S. C. Sivasamy M.. (2020). Genome-wide association studies in diverse spring wheat panel for stripe, stem, and leaf rust resistance. Front. Plant Sci. 11. doi: 10.3389/fpls.2020.00748 PMC7286347 32582265

[B49] Leath S. Bowen K. L. (1989). Effects of powdery mildew, triadimenol seed treatment Carolina, and triadimefon foliar sprays on yield of winter wheat in north. Phytopathology 79, 152–155. doi: 10.1094/Phyto-79-152

[B50] Leonova I. N. (2019). Genome-wide association study of powdery mildew resistance in Russian spring wheat (T. aestivum l.) varieties. Russ. J. Genet. 55, 1360–1374. doi: 10.1134/S1022795419110085

[B51] Li Y. Qiu L. Liu X. Zhang Q. Zhuansun X. Fahima T.. (2020). Glycerol-induced powdery mildew resistance in wheat by regulating plant fatty acid metabolism, plant hormones cross-talk, and pathogenesis-related genes. Int. J. Mol. Sci. 21, 673–692. doi: 10.3390/ijms21020673 PMC7013599 31968554

[B52] Liu N. Bai G. Lin M. Xu X. Zheng W. (2017). Genome-wide association analysis of powdery mildew resistance in U.S. winter wheat. Sci. Rep. 7, 1–11. doi: 10.1038/s41598-017-11230-z 28924158 PMC5603590

[B53] Liu Y. Liu Y. Zhang Q. Fu B. Cai J. Wu J.. (2018). Genome-wide association analysis of quantitative trait loci for salinity-tolerance related morphological indices in bread wheat. Euphytica 5, 1–11. doi: 10.1007/s10681-018-2265-5

[B54] Liu C. Sukumaran S. Jarquin D. Crossa J. Dreisigacker S. Sansaloni C.. (2020). Comparison of array- and sequencing-based markers for genome-wide association mapping and genomic prediction in spring wheat. Crop Sci. 60, 211–225. doi: 10.1002/csc2.20098

[B55] Liu Z. Sun Q. Ni Z. Nevo E. Yang T. (2002). Molecular characterization of a novel powdery mildew resistance gene Pm30 in wheat originating from wild emmer. Euphytica 123, 21–29. doi: 10.1023/A:1014471113511

[B56] Ma H. Liu Y. Zhao X. Zhang S. Ma H. (2022). Exploring and applying genes to enhance the resistance to fusarium head blight in wheat. Front. Plant Sci. 13. doi: 10.3389/fpls.2022.1026611 PMC9647131 36388594

[B57] Matika D. E. F. Loake G. J. (2014). Redox regulation in plant immune function. Antioxid. Redox Signal. 21, 1373–1388. doi: 10.1089/ars.2013.5679 24206122 PMC4158969

[B58] Maxwell J. J. Lyerly J. H. Cowger C. Marshall D. Brown-Guedira G. Murphy J. P. (2009). MlAG12: A triticum timopheevii-derived powdery mildew resistance gene in common wheat on chromosome 7AL. Theor. Appl. Genet. 119, 1489–1495. doi: 10.1007/s00122-009-1150-y 19760389

[B59] Mokry F. B. Buzanskas M. E. Mudadu M. D. A. Grossi A. Higa R. H. Ventura R. V.. (2014). Linkage disequilibrium and haplotype block structure in a composite beef cattle breed. BMC Genomics 15, S6. doi: 10.1186/1471-2164-15-S7-S6 PMC4243187 25573652

[B60] Mondal S. Sallam A. Sehgal D. Sukumaran Sivakumar Farhad M. Krishnan J. N.. (2021). “Advances in breeding for abiotic stress tolerance in wheat,” in. Genomic Des. Abiotic Stress Resistant Cereal Crops 71–103. doi: 10.1007/978-3-030-75875-2

[B61] Mourad A. M. I. Abou-Zeid M. A. Eltaher S. Baenziger P. S. Börner A. (2021a). Identification of candidate genes and genomic regions associated with adult plant resistance to stripe rust in spring wheat. Agronomy 11, 2585–2603. doi: 10.3390/agronomy11122585

[B62] Mourad A. M. I. Amin A. E. E. A. Z. Dawood M. F. A. (2021b). Genetic variation in kernel traits under lead and tin stresses in spring wheat diverse collection. Environ. Exp. Bot. 192, 104646. doi: 10.1016/j.envexpbot.2021.104646

[B63] Mourad A. Belamkar V. Baenziger P. S. (2020). Molecular genetic analysis of spring wheat core collection using genetic diversity, population structure, and linkage disequilibrium. BMC Genomics 21, 1–12. doi: 10.1186/s12864-020-06835-0 PMC7318758 32586286

[B64] Mourad A. M. I. Draz I. S. Omar G. E. Börner A. Esmail S. M. (2022a). Genome-wide screening of broad-spectrum resistance to leaf rust (Puccinia triticina eriks) in spring wheat (Triticum aestivum l.). Front. Plant Sci. 13. doi: 10.3389/fpls.2022.921230 PMC9258335 35812968

[B65] Mourad A. M. I. Eltaher S. Börner A. Sallam A. (2022b). Unlocking the genetic control of spring wheat kernel traits under normal and heavy metals stress conditions. Plant Soil. 484, 257–278. doi: 10.1007/s11104-022-05790-x

[B66] Mourad A. M. I. Sallam A. Belamkar V. Wegulo S. Bowden R. Jin Y.. (2018). Genome-wide association study for identification and validation of novel SNP markers for Sr6 stem rust resistance gene in bread wheat. Front. Plant Sci. 9. doi: 10.3389/fpls.2018.00380 PMC5881291 29636761

[B67] MQ S. XX Z. XY D. BQ S. (1987). Identification of physiologic race of erysiphe graminis f. sp. tritici. Sci. Agric. Sin. 20, 64–70.

[B68] Muhammad A. Hu W. Li Z. Li J. Xie G. Wang J.. (2020). Appraising the genetic architecture of kernel traits in hexaploid wheat using GWAS. Int. J. Mol. Sci. 21, 1–21. doi: 10.3390/ijms21165649 PMC7460857 32781752

[B69] Muhammad A. Li J. Hu W. Yu J. Khan S. U. Khan M. H. U.. (2021). Uncovering genomic regions controlling plant architectural traits in hexaploid wheat using different GWAS models. Sci. Rep. 11, 1–14. doi: 10.1038/s41598-021-86127-z 33762669 PMC7990932

[B70] Nigro D. Blanco A. Piarulli L. Signorile M. A. Colasuonno P. Blanco E.. (2022). Fine mapping and candidate gene analysis of Pm36, a wild emmer-derived powdery mildew resistance locus in durum wheat. Int. J. Mol. Sci. 23, 13659. doi: 10.3390/ijms232113659 36362444 PMC9657016

[B71] Noubissié E. Ngassoum M. B. Ali A. Castro-Georgi J. Donard O. F. X. (2017). Speciation of organometallic compounds of Hg, Sn, and Pb in the market garden soil in ngaoundéré (Cameroon). Euro-Mediterranean J. Environ. Integr. 2, 1–11. doi: 10.1007/s41207-017-0028-7

[B72] Paux E. Sourdille P. Salse J. Saintenac C. Choulet F. Leroy P.. (2008). A physical map of the 1-gigabase bread wheat chromosome 3B. Science (80). 322, 101–104. doi: 10.1126/science.1161847 18832645

[B73] Pearce S. Vazquez-Gross H. Herin S. Y. Hane D. Wang Y. Gu Y. Q.. (2015). WheatExp: An RNA-seq expression database for polyploid wheat. BMC Plant Biol. 15, 1–8. doi: 10.1186/s12870-015-0692-1 26705106 PMC4690421

[B74] Petersen S. Lyerly J. H. Worthington M. L. Parks W. R. Cowger C. Marshall D. S.. (2015). Mapping of powdery mildew resistance gene Pm53 introgressed from aegilops speltoides into soft red winter wheat. Theor. Appl. Genet. 128, 303–312. doi: 10.1007/s00122-014-2430-8 25425170

[B75] Qureshi N. Bariana H. Forrest K. Hayden M. Keller B. Wicker T.. (2017). Fine mapping of the chromosome 5B region carrying closely linked rust resistance genes Yr47 and Lr52 in wheat. Theor. Appl. Genet. 130, 495–504. doi: 10.1007/s00122-016-2829-5 27866228

[B76] Ross J. R. Nam K. H. D’Auria J. C. Pichersky E. (1999). S-adenosyl-L-methionine:salicylic acid carboxyl methyltransferase, an enzyme involved in floral scent production and plant defense, represents a new class of plant methyltransferases. Arch. Biochem. Biophys. 367, 9–16. doi: 10.1006/abbi.1999.1255 10375393

[B77] Salina E. A. Nesterov M. A. Frenkel Z. Kiseleva A. A. Timonova E. M. Magni F.. (2018). Features of the organization of bread wheat chromosome 5BS based on physical mapping. BMC Genomics 19, 132–141. doi: 10.1186/s12864-018-4470-y PMC5836826 29504906

[B78] Sallam A. Alqudah A. M. Baenziger P. S. Rasheed A. (2023). Editorial: Genetic validation and its role in crop improvement. Front. Genet. 13. doi: 10.3389/fgene.2022.1078246 PMC9846199 36685961

[B79] Sallam A. Alqudah A. M. Dawood M. F. A. Baenziger P. S. Börner A. (2019). Drought stress tolerance in wheat and barley: Advances in physiology, breeding and genetics research. Int. J. Mol. Sci. 20, 3137. doi: 10.3390/ijms20133137 31252573 PMC6651786

[B80] Sallam A. Mourad A. M. I. Hussain W. Stephen Baenziger P. (2018). Genetic variation in drought tolerance at seedling stage and grain yield in low rainfall environments in wheat (Triticum aestivum l.). Euphytica 214, 169. doi: 10.1007/s10681-018-2245-9

[B81] Singh R. P. Singh P. K. Rutkoski J. Hodson D. P. He X. Jørgensen L. N.. (2016). Disease impact on wheat yield potential and prospects of genetic control. Annu. Rev. Phytopathol. 54, 303–322. doi: 10.1146/annurev-phyto-080615-095835 27296137

[B82] Turuspekov Y. Ormanbekova D. Rsaliev A. Abugalieva S. (2016). Genome-wide association study on stem rust resistance in Kazakh spring barley lines. BMC Plant Biol. 16, 14–81. doi: 10.1186/s12870-015-0686-z PMC4895317 26821649

[B83] Utz H. (1997). PLABSTAT: A computer program for statistical analysis of plant breeding experiments. Version 2N. Stutgart, Germany: University of Hohenheim.

[B84] Wang N. Akey J. M. Zhang K. Chakraborty R. Jin L. (2002). Distribution of recombination crossovers and the origin of haplotype Blocks : The interplay of population history, recombination, and mutation. Am. J. Hum. Genet. 71, 1227–1234. doi: 10.1086/344398 12384857 PMC385104

[B85] Wang Q. Sun Y. Wang F. Huang P. C. Wang Y. Ruan X.. (2021). Transcriptome and oxylipin profiling joint analysis reveals opposite roles of 9-oxylipins and jasmonic acid in maize resistance to gibberella stalk rot. Front. Plant Sci. 12. doi: 10.3389/fpls.2021.699146 PMC8454893 34557211

[B86] Ward B. P. Brown-Guedira G. Kolb F. L. Van Sanford D. A. Tyagi P. Sneller C. H.. (2019). Genome-wide association studies for yield-related traits in soft red winter wheat grown in Virginia. PloS One 14, 1–28. doi: 10.1371/journal.pone.0208217 PMC6386437 30794545

[B87] Xue F. Ji W. Wang C. Zhang H. Yang B. (2012). High-density mapping and marker development for the powdery mildew resistance gene PmAS846 derived from wild emmer wheat (Triticum turgidum var. dicoccoides). Theor. Appl. Genet. 124, 1549–1560. doi: 10.1007/s00122-012-1809-7 22350087

[B88] Yin L. Zhang H. Tang Z. Xu J. Yin D. Zhang Z.. (2021). rMVP: A memory-efficient, visualization-enhanced, and parallel-accelerated tool for genome-wide association study. Genomics Proteomics Bioinf. 19, 619–628. doi: 10.1016/J.GPB.2020.10.007 PMC9040015 33662620

[B89] Zhou B. Benbow H. R. Brennan C. J. Arunachalam C. Karki S. J. Mullins E.. (2020). Wheat encodes small, secreted proteins that contribute to resistance to septoria tritici blotch. Front. Genet. 11. doi: 10.3389/fgene.2020.00469 PMC7235427 32477410

