# Supplementary figures and images for: Novel genomic regions on chromosome 5B controlling wheat powdery mildew seedling resistance under Egyptian conditions
Source: Front Plant Sci. 2023 May 10;14:1160657. doi: 10.3389/fpls.2023.1160657 (PMC10208068; doi:10.3389/fpls.2023.1160657)

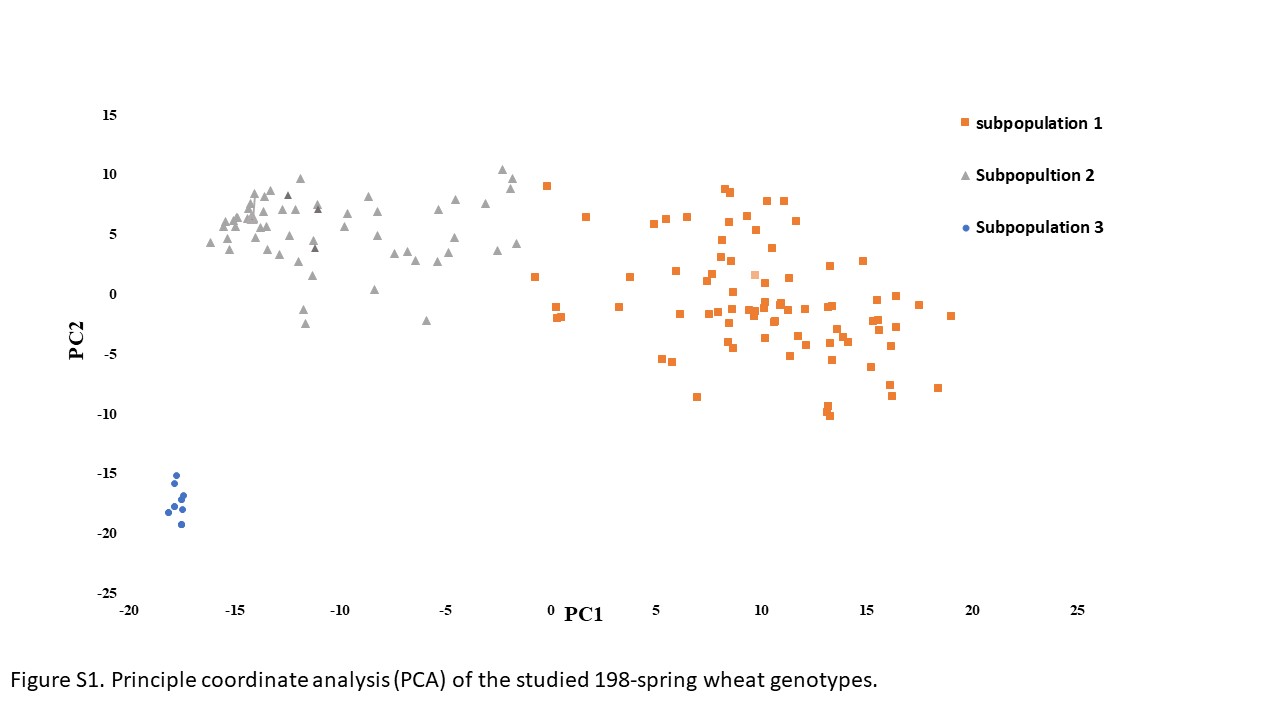

Supplement: Supplementary file 1 [file Image_1.jpeg]

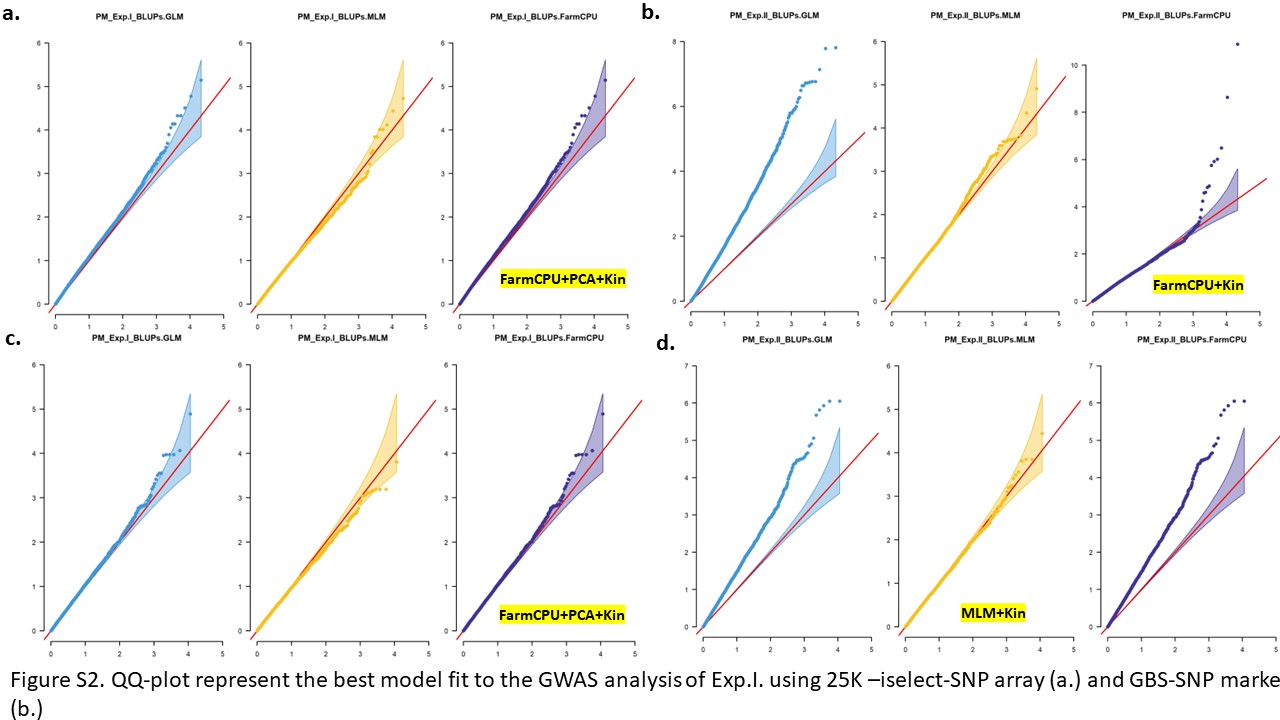

Supplement: Supplementary file 2 [file Image_2.jpeg]

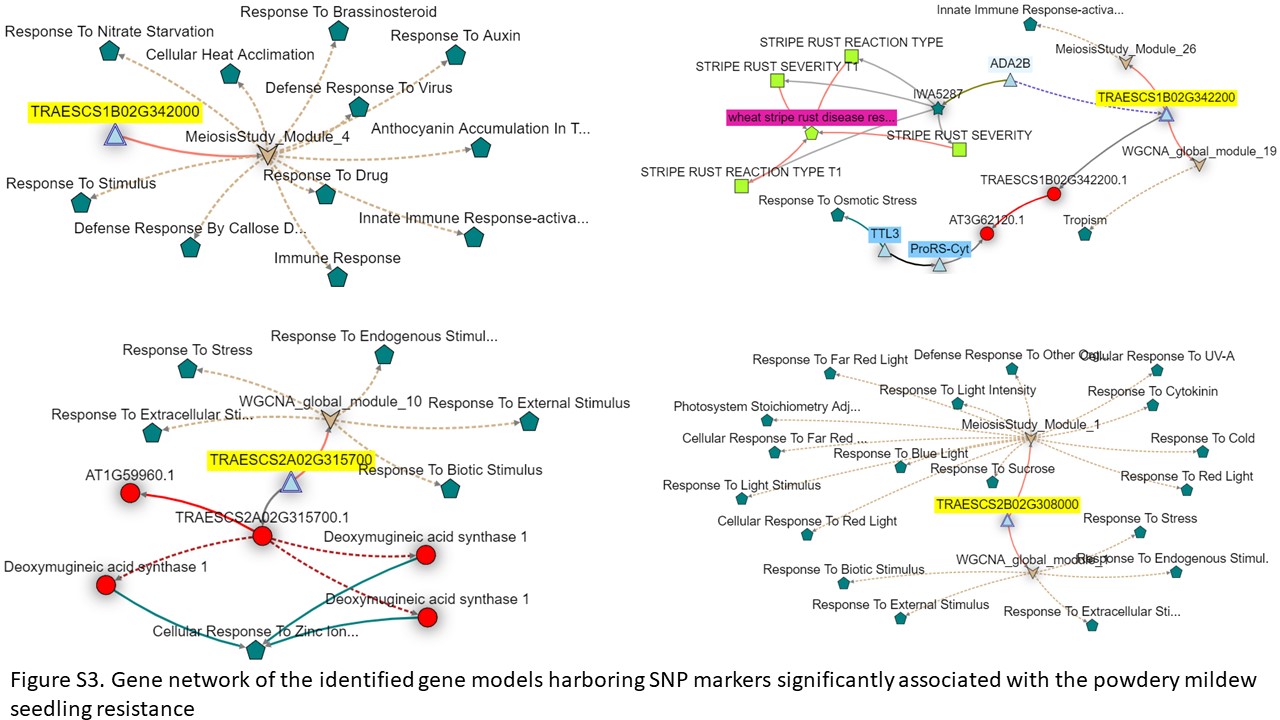

Supplement: Supplementary file 3 [file Image_3.jpeg]

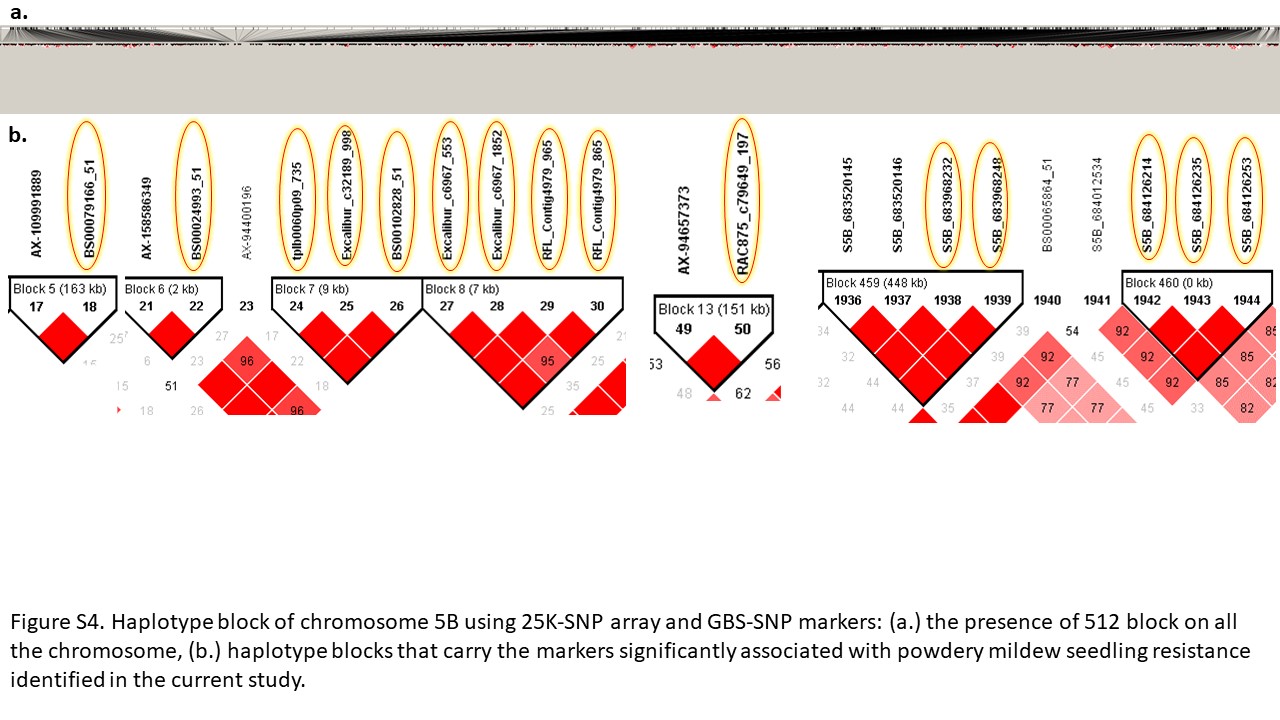

Supplement: Supplementary file 4 [file Image_4.jpeg]

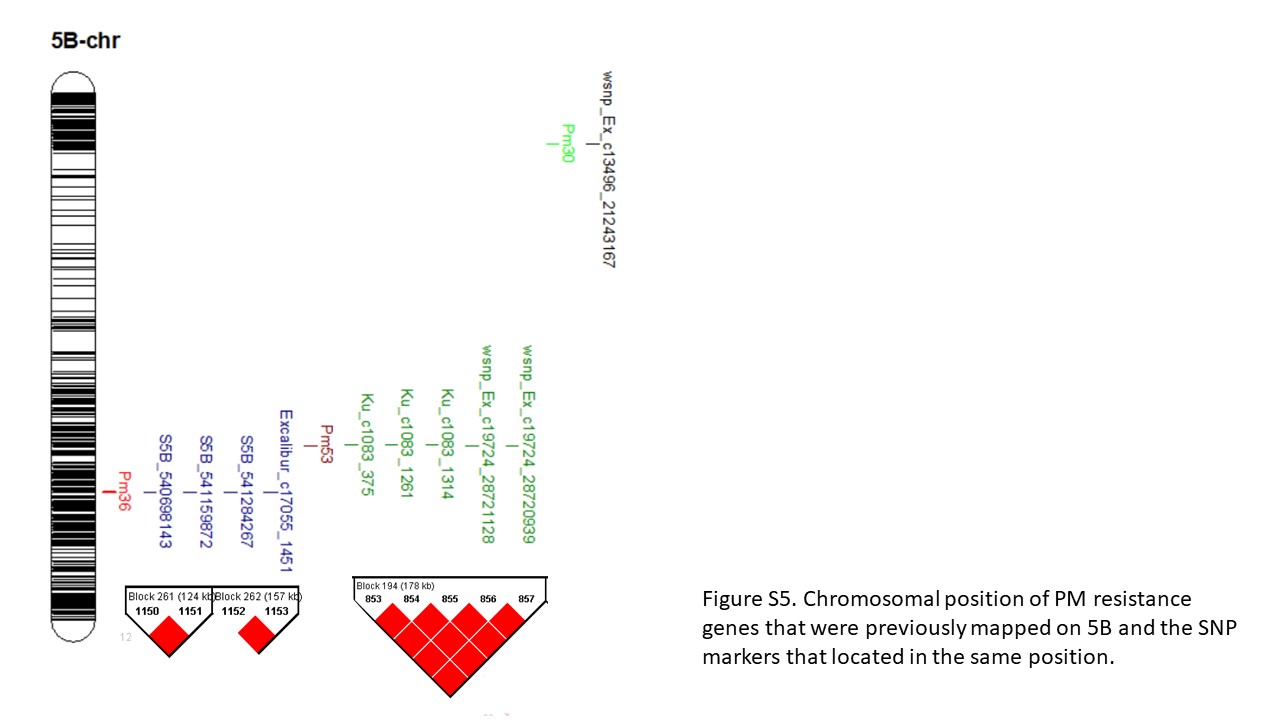

Supplement: Supplementary file 5 [file Image_5.jpeg]
